# Non-invasive risk stratification of metabolic dysfunction-associated steatotic liver disease and liver fibrosis in adults with type 1 diabetes

**DOI:** 10.3389/fendo.2026.1794005

**Published:** 2026-03-11

**Authors:** Manuel Ramón García-Sáenz, Marilu Cervantes-Maldonado, Luis Angel López-Cruz, Eduardo Salif Luna-Avila, Paulo César Gete Palacios, Cristina Martínez-Berdeja, Omar Jaime-Leal, Aldo Ferreira-Hermosillo

**Affiliations:** 1 Servicio de Endocrinología, Hospital de Especialidades “Dr. Bernardo Sepúlveda”, Centro Médico Nacional Siglo XXI, Instituto Mexicano del Seguro Social, Mexico City, Mexico; 2 Gastroenterology Consultant, Hospital Grinker, Torreón, Mexico; 3 Unidad de Investigación Médica en Enfermedades Endocrinas, Centro Médico Nacional Siglo XXI, Instituto Mexicano del Seguro Social, Mexico City, Mexico

**Keywords:** body composition, elastography, fatty liver, liver fibrosis, MASLD, Mexico, type 1 diabetes

## Abstract

**Background:**

Metabolic dysfunction-associated steatotic liver disease (MASLD) is an emerging comorbidity among patients with type 1 diabetes (T1D) that can potentially increase liver and cardiovascular outcomes. The aim of this study was to evaluate hepatic steatosis and to characterize non-invasive markers of MASLD and liver fibrosis risk in adults with T1D.

**Methods:**

A cross-sectional, observational, single-center study was conducted from 2023–2024. The sample was adults with T1D at a tertiary hospital in Mexico. We recorded clinical, body-composition, and laboratory data. Hepatic steatosis was assessed by ultrasound, and non-invasive indices [Hepatic steatosis index (HSI), fatty liver index (FLI), and Fibrosis-4 (FIB-4)] and 2D shear-wave elastography (2D-SWE) were used to stratify the risk of MASLD and liver fibrosis. Bivariable and correlation analyses were performed.

**Results:**

Sixty-five adults were included (61.5% women; age 34 ± 10 years; T1D duration 20 [15–28] years; dyslipidemia 45%; overweight/obesity 51%; HbA1c 8.8 ± 2.0%. Ultrasound-compatible hepatic steatosis was observed in 25% (n=16). Noninvasive indices identified a higher proportion of individuals at increased risk for MASLD and fibrosis. HSI flagged 52% at risk and FLI 25% at high risk. Mean FIB-4 was 0.64 ± 0.36; 20% had fibrosis stage 2–3 and one stage 4–5. Elastography stiffness averaged 5.9 ± 0.69 kPa; 5% ≥7–8 kPa. MASLD was associated with higher stiffness, FIB-4, AST and lower platelets (all p<0.05; HSI p=0.06). Visceral fat correlated with HSI (r=0.525) and FLI (r=0.505) and inversely with FIB-4 (r=−0.261).

**Conclusions:**

Non-invasive tools allow the identification of adults with T1D at increased risk for steatotic liver disease and fibrosis; however, these findings should be interpreted as risk stratification rather than definitive diagnosis.

## Introduction

Metabolic dysfunction-associated steatotic liver disease (MASLD) is recognized as a clinically relevant comorbidity associated with adverse hepatic, cardiovascular, and renal outcomes. Traditionally, MASLD has been closely linked to metabolic conditions such as hyperglycemia, dyslipidemia and abdominal obesity, and insulin resistance, and its global prevalence has risen in parallel with the growing burden of metabolic disease worldwide (1).

Type 1 diabetes (T1D) is a disease characterized by an absolute deficiency of insulin secondary to autoimmune destruction of pancreatic beta cells. Although historically considered metabolically distinct from type 2 diabetes (T2D), individuals with T1D are not exempt from metabolic complications. Recent evidence suggests a prevalence of MASLD in approximately 22.2% of adults with T1D; moreover, significant fibrosis (> F2) is observed in 13.2% and advanced fibrosis (> F3) in 5.12% of them (2). That is clinically meaningful given the relatively young age and long-life expectancy of many individuals with T1D.

The development of MASLD is related to a persistent inflammatory state and is favored by lipotoxicity due to the accumulation of ceramides and free cholesterol, which in turn activates pathways such as the NF-κB pathway as well as the NLRP3 inflammasome (3). In individuals with T1D, MASLD risk may be influenced by distinct and disease-specific factors, including lifelong exposure to exogenous insulin, peripheral hyperinsulinemia with relative portal hypoinsulinemia, chronic hyperglycemia, marked glycemic variability, recurrent hypoglycemia, and progressive alterations in body composition. Even in the absence of classical insulin resistance, these factors may promote hepatic *de novo* lipogenesis, oxidative stress, and lipid accumulation through alternative metabolic pathways (4, 5).

Given the association of MASLD and adverse clinical outcomes, international guidelines recommend screening individuals with metabolic risk factors through the use of clinical tools and non-invasive indices (aspartate aminotransferase (AST)/alanine transaminase (ALT) levels, Fibrosis-4 index (FIB-4), hepatic steatosis index (HSI), and fatty liver index (FLI)) and, in selected cases, imaging methods or invasive procedures (6). The combined use of scales and imaging studies offers an accessible and low-cost method for identifying patients at risk of liver fibrosis, facilitating early preventive and therapeutic interventions (6, 7). Nevertheless, a specific guidance for MASLD screening in individuals with T1D remains limited.

Most non-invasive indices currently used to stratify MASLD, and liver fibrosis risk were developed and validated in T2D or cohorts with metabolic diseases. However, their diagnostic performance in T1D remains uncertain, as these indices rely on anthropometric, biochemical, and hematological variables (such as transaminase levels, platelet counts, and measures of adiposity) that may behave differently in the patients with T1D. Emerging evidence suggests that standard cut-off values may underestimate MASLD risk in T1D, highlighting the need for population-specific evaluation and calibration (8).

Data on MASLD prevalence and risk stratification in adults with T1D from Latin America are scarce. Regional differences in glycemic control, cardiometabolic risk profiles, healthcare access, and socioeconomic factors may influence both disease prevalence and clinical presentation. Accordingly, the aim of this study was to evaluate hepatic steatosis and to characterize non-invasive markers of MASLD and liver fibrosis risk in a well-characterized cohort of adults with T1D from Mexico, using a multimodal approach that integrates biochemical indices, body composition assessment, ultrasound, and two-dimensional shear-wave elastography (2D-SWE).

## Materials and methods

### Study design

This was a cross-sectional, observational study. Clinical history and glycemic control variables were obtained from retrospective review of medical records, while liver ultrasound and elastography assessments were performed at the time of study inclusion following a standardized protocol.

### Population

**Figure 1:**
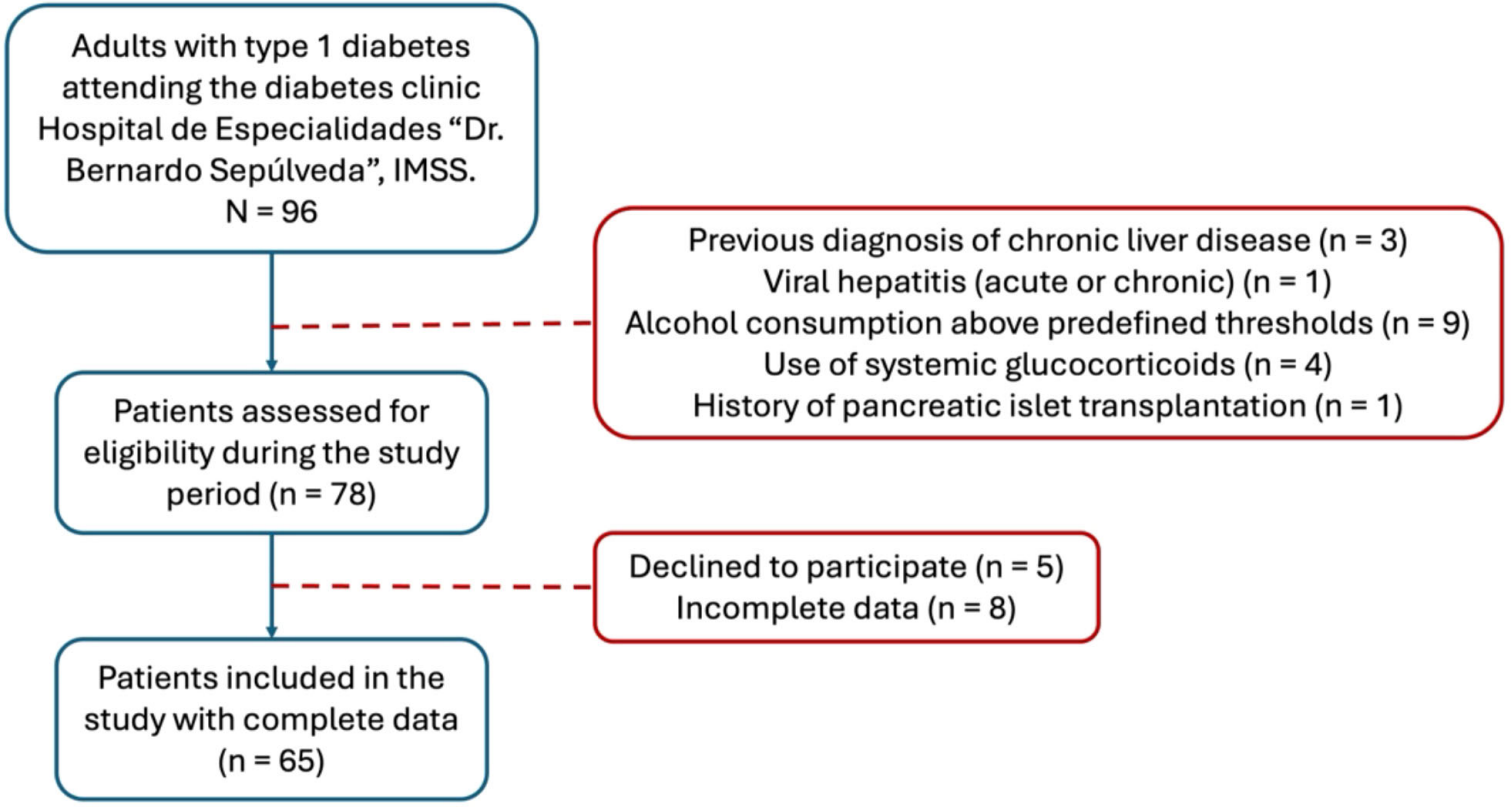
Flow diagram of participant inclusion and exclusion. Adults with type 1 diabetes were screened during routine outpatient follow-up between 2023-2024. Clinical and glycemic variables were collected retrospectively, while liver ultrasound and elastography assessments were performed prospectively. All participants included in the study met predefined reliability criteria for elastography measurements.

The study sample consisted of patients with T1D, including men and women older than 18 years who were treated at the diabetes clinic of the Hospital de Especialidades “Dr. Bernardo Sepúlveda” of the Centro Médico Nacional Siglo XXI (IMSS, Mexico) from 2023–2024 (**Figure 1**). Patients with a previous diagnosis of chronic liver disease, acute or chronic viral liver infection, who were using glucocorticoids, who had a history of pancreatic islet transplantation, or who reported alcohol consumption >20 g/day in women and >30g/day in men were excluded. All patients signed informed consent forms.

The study was conducted in accordance with the Declaration of Helsinki and approved by the Local Review Board and Ethics Committee of the Hospital de Especialidades Centro Médico Nacional Siglo XXI, Instituto Mexicano del Seguro Social (protocol code R-2024-3601-062, date of approval February 2024).

### Definition of type 1 diabetes

T1D was defined based on a clinical diagnosis established by an endocrinologist, including disease onset at a young age, lifelong insulin dependence from diagnosis, autoantibody test (Anti-GAD65) and low C-peptide levels (<0.95 ng/ml).

### Clinical variables

The following data were recorded: age, sex, comorbidities (hypothyroidism, arterial hypertension, and dyslipidemia), duration of diabetes, insulin dose, body mass index (BMI), and waist and hip circumferences. The body composition was evaluated via multifrequency bioimpedance (InBody 120; Biospace Co., Ltd., Seoul, Korea), yielding the percentage of total body fat and the visceral fat score.

Medication review included the current or recent (≤6 months) use of sodium-glucose cotransporter-2 (SGLT2) inhibitors, glucagon-like peptide-1 receptor agonists, and statins.

### Biochemical variables

Glycated hemoglobin (HbA1c), AST, and ALT levels, platelet counts, and gamma-glutamyl transferase (GGT) and triglyceride levels were obtained according to routine control protocols. For the blood chemistry and glycated hemoglobin tests, an automated COBAS c503 photometry-based analyzer (Roche Diagnostics, Mannheim, Germany) was used; for hematic biometry, a Sysmex XN-1000 hematology analyzer (Sysmex Corporation, Kobe, Japan) and flow cytometry were used.

### MASLD diagnosis

Hepatic steatosis was defined by ultrasonographic findings compatible with fatty liver. Elevated transaminase levels were considered biochemical markers of liver injury but no diagnostic criteria for MASLD.

Non-invasive indices (HSI, FLI) were used to estimate the risk of MASLD, and FIB-4 and 2D-SWE were used for fibrosis risk stratification. According to current European Association for the Study of the Liver (EASL) and the American Association for the Study of Liver Diseases (AASLD) recommendations (6, 9), MASLD requires objective evidence of hepatic steatosis; therefore, this study was designed to evaluate non-invasive risk stratification rather than definitive MASLD prevalence.

### Elastography

Hepatic stiffness was measured by two-dimensional shear wave elastography (2D-SWE) with a LOGIQ E9 device (GE Healthcare, Chicago, IL, USA) and a convex transducer after the patients had fasted for > 4 h. The patients were placed in the supine position with the right arm abducted. More than 10 measurements were made in the right liver lobe at a depth of 1.5–4 cm below the capsule while avoiding the vessels. The median hepatic stiffness is expressed in kilopascals (kPa). Reliability was defined as an IQR/median ratio <30% (10, 11). No participants were excluded due to failure to meet elastography reliability criteria.

All ultrasound and elastography examinations were performed by a single experienced radiologist who was blinded to clinical and laboratory data. Interobserver variability was not assessed, as all measurements were obtained by the same operator.

### Calculated indices

- FLI: [0.953 × ln(triglycerides) + 0.139 × BMI + 0.718 × ln(GGT) + 0.053 × waist circumference - 15.745]. Interpretation: <30, low risk; 30–60, intermediate risk; > 60, high risk (12).
- FIB-4: [(Age × AST)/(Platelets × √ALT)]. Interpretation: <1.45, fibrosis stage 0–1; 1.45–3.25, fibrosis stage 2–3; > 3.25, fibrosis stage 4–5 (13).
- HSI: [8 × ALT/AST + BMI + 2 (if had diabetes) + 2 (if female)]. Interpretation: <30, very low risk; 30–36 intermediate risk; > 36, high risk (14).

### Statistical analysis

Continuous variables are presented as mean ± standard deviation (SD) or median (IQR). Categorical variables are shown as n (%) and compared using Student’s t-test or Mann-Whitney U test and χ^2^ or Fisher’s exact test as appropriate. Pearson or Spearman correlations assessed linear associations. A multivariable logistic regression was used to identify independent factors associated with MASLD. To minimize the risk of overlifting given the sample size, only variables showing strong associations in bivariable analyses were entered into the multivariable models. A linear regression model assessed determinants of liver stiffness (kPa). Model assumptions (collinearity, linearity, residual normality) were verified and model discrimination was assessed using the area under receiver operating characteristic curve (AUC), and calibration was evaluated with the Hosmer-Lemeshow test. Two-tailed p < 0.05 was considered significant (SPSS v25).

### Sample size and power analysis

This study was designed as a pilot cross-sectional analysis; therefore, no *a priori* sample-size calculation was performed. *Post-hoc* calculations indicated that with n=65 (16 with MASLD vs 49 without MASLD) and α = 0.05, the study had ~80% power to detect large between-group differences (Cohen’s d ~ 0.78) and correlations [r] ≥ 0.33. Smaller effects are likely underpowered; given the exploratory nature of the study, results from multivariable analyses should be interpreted as hypothesis-generating.

## Results

### Baseline clinical and metabolic characteristics of the study population

Sixty-five patients were included, 61.5% of whom were women (n = 40), and the mean age was 34 ±10 years. The most frequent comorbidity was dyslipidemia (45%, n = 29). The duration of T1D was > 20 years in 60% (n = 39) of the patients, with a median of 20 (IQR 15–28) years. The mean HbA1c level was 8.8 ± 2.0%. A total of 51% (n = 33) of the patients had overweight or obesity. The percentage of total body fat was 32.2 ± 5.5%, and the visceral fat score was 16.8 ± 2.9. The mean insulin dose was 0.67 ± 0.31 U/kg/day (**Table 1**).

**Table 1:** Baseline clinical and metabolic characteristics of adults with T1D (n=65).

Variable	Value
Age, years	34 ± 10
Female sex, n (%)	40 (61.5)
Diabetes duration, years	20 (15-28)
Hypothyroidism; n (%)	6 (9.2)
Hypertension, n (%)	4 (6.2)
Dyslipidemia, n (%)	29 (44.6)
Body Mass Index, kg/m^2^	25 (22–26.7)
Waist Circumference, cm	90 ± 13
Hip Circumference, cm	105 ± 11
Total Body Fat, %	32.2 ± 5.5
Visceral Fat Score	16.8 ± 2.9
Glycated Hemoglobin, %	8.8 ± 2.0
Insulin Dose, U/kg/day	0.67 ± 0.31
Aspartate Transaminase, U/L	24 ± 13
Alanine Transaminase, U/L	25 ± 17
Platelets, *10^9^/L	294 ± 74
Overweight or Obesity, n (%)	33 (50.8)
Metabolic Syndrome*, n (%)	55 (84.6)

Data are presented as mean ± standard deviation or median (interquartile range), according to distribution.

*Metabolic syndrome defined according to NCEP ATP III criteria.

### Prevalence of ultrasound-compatible hepatic steatosis

Ultrasound-compatible hepatic steatosis was identified in 25% (n = 16) of participants. Based on non-invasive indices, a higher proportion of patients were classified as being at increased risk for MASLD or fibrosis.

### Risk stratification by non-invasive indices

According to the indices, the HSI classified 52% (n = 34) of the patients as being at risk, and the FLI classified 25% (n = 16) of the patients as being at high risk. In the fibrosis evaluation, the mean FIB-4 was 0.64 ±0.36; 14% (n = 9) of the patients had stage 2–3 fibrosis, and two patients had stage 4–5 fibrosis. Elastography revealed a mean hepatic stiffness value of 5.9 ±0.69 kPa; only 5% (n = 3) of the patients had values ≥ 7–8 kPa, suggesting significant fibrosis (≥ F2), according to the cutoff points for 2D-SWE (10, 11). (**Table 2**).

**Table 2:** Risk stratification using non-invasive indices for MASLD and liver fibrosis.

Index	Category	n (%)
Hepatic Steatosis Index (HSI)	Low probability (<30)	7 (11)
	Intermediate probability (30-36)	24 (37)
	High probability (>36)	34 (52)
Fatty Liver Index (FLI)	Low risk (<30)	30 (46)
	Intermediate risk (30-60)	19 (29)
	High risk (>60)	16 (25)
Fibrosis-4 Index (FIB-4)	Low risk (<1.45)	54 (83)
	Intermediate risk (1.45-3.25)	9 (14)
	High risk (>3.25)	2 (3)
2D-SWE liver stiffness (kPa)	≥7–8 kPa	3 (5)

MASLD and fibrosis categories represent risk stratification based on validated cut-off values. Liver stiffness ≥8 kPa suggests significant fibrosis (≥F2).

### Bivariable associations according to MASLD status

Bivariable analysis revealed significant differences in the elastography-derived hepatic stiffness, FIB-4 values, AST levels, ALT levels and platelet counts between patients with and without MASLD. The elastography stiffness values were 6.2 ±0.56 and 5.8 ±0.7 kPa, respectively (p = 0.03). The FIB-4 values were 0.84 ±0.5 and 0.58 ±0.7, respectively (p = 0.011). The AST concentrations were 34 ±21 and 21 ±6 U/L, respectively (p = 0.001). Compared with the non-MASLD group, the MASLD group had a lower platelet count (261,000 ±71,000 vs. 305,000 ±73,000; p = 0.041) and a higher HSI (38.2 ±6.6 vs. 35.5 ±4.2; p = 0.06). However, the last one was not significative. (**Table 3**).

**Table 3:** Bivariate analysis of clinical and metabolic characteristics in patients with T1D stratified by MASLD status.

Variable	No MASLD (n=49)	MASLD (n=16)	p
Age, years	33 ± 11	39 ± 9	0.058
Female, n (%)	29 (59)	11 (69)	0.56
Diabetes duration, years	21 (14–28)	20 (16–28)	0.86
Dyslipidemia, n (%)	19 (39)	10 (63)	0.097
Body Mass Index, kg/m^2^	25 (22–27)	25 (19.9–27.4)	0.86
Total Body Fat, %	32.9 ± 5.0	29.8 ± 6.3	0.089
Visceral Fat Score	17.2 ± 2.7	15.6 ± 3.1	0.087
Glycated Hemoglobin, %	8.8 ± 2.0	8.9 ± 1.8	0.951
**Aspartate Transaminase, U/L**	**21 ± 6**	**34 ± 21**	**0.021**
**Alanine Transaminase, U/L**	**19 ± 5**	**44 ± 26**	**0.002**
Platelets, *10^9^/L	**305 ± 73**	**261 ± 71**	**0.041**
HSI	35.5 ± 4.2	38.2 ± 6.6	0.06
FLI	36.0 ± 22.6	34.6 ± 31.3	0.867
**Elastography, kPa**	**5.8 ± 0.7**	**6.2 ± 0.56**	**0.030**
**FIB-4**	**0.58 ± 0.28**	**0.84 ± 0.5**	**0.011**
Statin use, n (%)	28 (57.1)	7 (43.8)	0.351
iSGLT2 use, n (%)	23 (46.9)	7 (43.8)	0.824
GLP-1 agonist receptor use, n (%)	2 (4.1)	2 (12.5)	0.224

Bold p-values indicate statistical significance (p < 0.05). Data are presented as mean ± standard deviation, median (interquartile range). MASLD, metabolic dysfunction-associated steatotic liver disease; HSI, hepatic steatosis index; FLI, fatty liver index; FIB-4, fibrosis-4 index; iSGLT2, sodium-glucose transport 2 inhibitor; GLP-1, glucagon-like peptide type 1.

The visceral fat score was negatively correlated with the FIB-4 (r = -0.261; p = 0.036) and AST level (r = -0.311; p = 0.12) and positively correlated with the HSI (r = 0.525; p <0.001) and FLI (r = 0.505; p <0.001).

### Multivariable predictors of MASLD

In multivariable logistic regression including AST, platelet count, diabetes duration, visceral fat score, HbA1c, BMI, age, sex, and use of SGLT2 inhibitors, statins, and GLP-1 receptor agonists, only AST and platelets remained independently associated with MASLD. Higher AST levels were strongly associated with increased odds of MASLD (aOR=1.38; 95% CI 1.15-1.67; p = 0.001), whereas higher platelet counts showed an inverse association (aOR = 0.97; 95% CI 0.96-0.99; p = 0.012). Sex showed a non-significant trend toward association (aOR = 10.77; 95% CI 0.87-132.93; p = 0.064), but confidence intervals were wide and included the null value. Other variables, including HbA1c, BMI, age, diabetes duration, visceral fat score, and cardiometabolic medications, were not independently related to MASLD in the adjusted model. The model showed excellent discrimination (AUC = 0.94; 95% CI 0.89-1.0) and good calibration (Hosmer-Lemeshow χ^2^(7) = 2.81; p = 0.9).

### Determinants of liver stiffness assessed by 2D-SWE

In the multivariable linear regression model with liver stiffness (kPa) as the dependent variable, none of the predictors showed a robust independent association with stiffness. Although age had a statistically significant coefficient (β = 0.028 kPa per year; 95% CI 0.002-0.055; p = 0.036), the overall model was not significant (F(11,53) = 0.87; p = 0.58; R2 ≈ 0.15), and the effect sizes were small. Accordingly, no clinically meaningful determinants of liver stiffness could be identified in this cohort.

## Discussion

This study does not aim to establish the true prevalence of MASLD, as histological confirmation and advanced quantitative imaging were not available. Instead, our findings should be interpreted as a non-invasive risk stratification of steatotic liver disease and fibrosis in adults with type 1 diabetes, aligned with current international recommendations.

In this cohort of patients with T1D, we identified a 25% compatible with ultrasound steatotic liver disease, which is slightly higher than that reported in a previous meta-analysis, which yielded an estimate of 22% (2). This is an important finding considering that the population in this study was relatively young (average age of 34 years) and consisted mostly of women (61.5%), which in principle could be associated with a lower risk of liver disease.

### T1D-specific mechanisms

The reported prevalence suggests that, beyond classical metabolic risk factors, additional mechanisms specific to T1D may contribute to MASLD development. In contrast to T2D, where insulin resistance is the primary driver, MASLD in T1D appears to result from a complex interplay between overweight, visceral adiposity, dyslipidemia, and sustained hyperglycemia, in a metabolic context shaped by exogenous insulin exposure and glycemic variability.

Compared with previous T1D cohorts, our study includes younger participants and combines non-invasive indices with 2D-SWE and body-composition analysis, providing complementary risk stratification. Prior meta-analyses have rarely explored visceral fat or bioimpedance-derived metrics in this population.

Multiple studies have reported that the duration of diabetes, dyslipidemia, and suboptimal glycemic control are independent risk factors for MASLD in patients with T1D. In our cohort, 60% of the patients had a disease duration of more than 20 years, and 45% had dyslipidemia; these findings are consistent with those described in European and American registries (2, 8). The mean HbA1c level of 8.8%, reflecting poor glycemic control, is similar to that (8.7%) reported in the National Registry of Type 1 Diabetes in Mexico (15), which strengthens the hypothesis that chronic exposure to hyperglycemia could play a key role in the pathophysiology of MASLD in this group.

In contrast to type 2 diabetes (T2D), in which insulin resistance is the central mechanism, in T1D patients, the risk of MASLD appears to be mediated by the combination of overweight, excess visceral adiposity, dyslipidemia and sustained hyperglycemia, while the development of a similar form of insulin resistance likely also contributes. These factors create an environment that favor liver lipotoxicity, oxidative stress and chronic inflammation, leading to the progression from steatosis to fibrosis (4, 5). In fact, the visceral fat score in our cohort was significantly correlated with liver disease risk indices (HSI and FLI), underscoring the importance of assessing body composition beyond the BMI in this population.

### Non-invasive indices in T1D

Non-invasive indices such as HSI and FLI were originally validated in T2D and general populations; therefore, their performance in T1D may differ, highlighting the need for disease-specific thresholds. In our study, the HSI and the FLI classified 52% and 25% of the population, respectively, as being at high risk. However, the applicability of these indices to T1D patients remains inconclusive. For example, Mertens et al. reported that an FLI > 60 missed up to 45% of confirmed MASLD patients among those with T1D, suggesting a cutoff point of > 30 for screening (16). These data highlight the need to adapt these indices to the pathophysiological characteristics of T1D and validate them in T1D patients.

### Fibrosis assessment

Fibrosis evaluation 2D-SWE performed with a LOGIQ E9 device, revealed a mean stiffness value of 5.9 kPa, and only 5% of the patients were in the range suggestive of significant fibrosis. The cutoff points for SWE differ from those for transient elastography (VCTE). Previous meta-analyses defined thresholds of ~7–8 kPa for F2, ~9–10 kPa for > F3 and > 11–12 kPa for F4 (10, 11, 17). Therefore, most of our cohort were in the nonsignificant range. However, the FIB-4 values classified 20% of the patients as being at intermediate risk; this discrepancy may be due to the influence of transaminase levels and platelet counts on the FIB-4 or the lack of specific cutoff points for T1D. However, the lack of liver biopsy data prevents drawing definitive conclusions about the diagnostic precision of each tool.

The presence of MASLD in a relatively young population with T1D and poor glycemic control has relevant clinical implications. First, it suggests the need to systematically incorporate liver screening in the follow-up of these patients, like the recommendations for T2D patients. Second, although the proportion of patients with fibrosis was low, it was not negligible, which necessitates the implementation of early preventive strategies, since progression to cirrhosis and hepatocellular carcinoma is possible even in young patients. Another relevant aspect is the possible interaction between MASLD and cardiovascular risk. Previous studies have shown that MASLD increases the risk of cardiovascular events in patients with T1D, regardless of the degree of glycemic control (18). Given that the main cause of mortality in T1D patients is cardiovascular disease, the identification of MASLD could become an additional risk stratification tool, reinforcing the need for the early detection of this disease.

Several parameters showed large standard deviations in participants with MASLD, reflecting substantial biological heterogeneity. Extreme values were retained to avoid selection bias, and non-parametric statistical methods were applied when appropriate. This variability may represent different stages of disease evolution within the cohort.

### Multivariable models and overfitting

In our adjusted analysis, AST and platelet count emerged as the only independent predictors of MASLD, which is consistent with biochemical patterns linked to steatotic liver disease and early fibrotic remodeling. The inverse association with platelets likely reflects the known relationship between thrombocytopenia and more advanced liver injury, even though most patients in this cohort remained within non-cirrhotic ranges. The trend for sex did not reach statistical significance and was characterized by wide confidence intervals, probably reflecting the small number of MASLD cases and limited statistical power, so this finding should be interpreted with caution.

Importantly, we did not identify robust independent determinants of liver stiffness by 2D-SWE in multivariable linear models. Although age showed a modest association with stiffness, the overall model fit was poor and explained only a small proportion of the variance. Together with the excellent discrimination but optimistic performance of the logistic model (AUC 0.94 in a relatively small sample), these results underline the exploratory nature of our analyses and reinforce the need for larger, histologically validated T1D cohorts to define reliable, T1D-specific thresholds for non-invasive stratification.

### Limitations and strengths

The main limitation of the study was the lack of histological validation. Consequently, non-invasive tools could not be calibrated against the biopsy gold standard. Future multicenter studies should incorporate paired biopsy-elastography comparisons to define T1D-specific thresholds. In addition, the single-center design and small sample size restrict generalizability; this, these results should be interpreted as exploratory. Likewise, an unconventional SWE technique was used to evaluate liver stiffness. SWE has advantages over VCTE, such as availability in conventional ultrasound scanners, real-time visualization, and a lower failure rate in obese patients (19); however, it also has several limitations, such as operator dependence, influence from acute inflammation, congestion or cholestasis on stiffness, and interplatform variability (20). In our study, it was not possible to quantify steatosis through controlled attenuation parameters (e.g., CAP), which would have allowed a more precise evaluation of steatosis. The small sample size and the single-center design limit the generalizability of the findings.

Among the strengths of this study is the inclusion of a well-characterized cohort of patients with T1D in Mexico, a group that is little studied in this context. In addition, a multimodal approach with biochemical indices, ultrasound and elastography was used, which provides a comprehensive view of the spectrum of liver disease in this population. The integration of body composition analysis with biochemical and imaging tools, and the generation of novel data from a Latin American population that is underrepresented in the literature.

The inverse correlation between visceral fat and FIB-4 (r ~ -0.26) may reflect the age- and platelet-dependent structure of FIB-4 rather than a biological inverse relationship. In T1D, low body weight, partial lipodystrophy, and decoupling of adiposity from transaminase activity could mask fibrosis risk. This underscores the need for T1D-specific calibration of composite indices integrating adiposity, enzymatic, and stiffness parameters.

The findings of this study open several lines of research. First, multicenter studies with larger sample sizes and histological validation are needed to define the real utility of noninvasive MASLD indices in patients with T1D. Second, longitudinal evaluations of the progression of MASLD in this population, as well as its relationship with micro- and macrovascular complications, would be useful. Finally, research on emerging therapies for MASLD, which are currently under evaluation in T2D patients, should be extended to T1D, especially considering that strict glycemic control alone, although necessary, may not be sufficient to prevent the progression of liver disease.

## Conclusions

MASLD is an emerging and clinically relevant complication in patients with T1D who are at risk of progression to fibrosis and cirrhosis. Early diagnosis and timely management of the disease are essential to reduce adverse hepatic and cardiovascular outcomes. In this cohort, noninvasive methods (HSI, FLI, FIB-4, and elastography-based hepatic stiffness) showed potential utility in identifying patients at risk, although this utility needs to be further validated in this population. The lack of liver biopsy data limits definitive interpretations of the results, which nevertheless suggest a particular pattern of disease in patients with T1D.

The present pilot study achieved 80% statistical power only for large effects; consequently, findings should be confirmed in larger, histologically validated cohorts. Nevertheless, our data highlight the potential of integrated non-invasive assessment for early MASLD detection in adults with T1D.

MASLD screening should be incorporated into the comprehensive care of patients with T1D. Multicenter studies with histological confirmation are needed to establish specific diagnostic criteria and explore emerging therapies for managing this comorbidity.

## Data Availability

The raw data supporting the conclusions of this article will be made available by the authors, without undue reservation.
